# Evaluating Anesthesia Practices, Patient Characteristics, and Outcomes in Electroconvulsive Therapy: A Two-Year Retrospective Study

**DOI:** 10.3390/jcm13206253

**Published:** 2024-10-19

**Authors:** Bogdan Ioan Vintilă, Claudia Elena Anghel, Mihai Sava, Alina-Simona Bereanu, Ioana Roxana Codru, Raul Stoica, Alexandra-Maria Vulcu Mihai, Andreea-Maria Grama, Alina Camelia Cătană, Adrian Gheorghe Boicean, Adrian Hașegan, Alin Mihețiu, Ciprian-Ionuț Băcilă

**Affiliations:** 1 Faculty of Medicine, Lucian Blaga University of Sibiu, 550024 Sibiu, Romania; bogdan.vintila@ulbsibiu.ro (B.I.V.); claudia.anghel@ulbsibiu.ro (C.E.A.); ioanaroxana.bera@ulbsibiu.ro (I.R.C.); alinabrabete@yahoo.com (A.C.C.); adrian.boicean@ulbsibiu.ro (A.G.B.); adrian.hasegan@ulbsibiu.ro (A.H.); alin.mihetiu@ulbsibiu.ro (A.M.); ciprian.bacila@ulbsibiu.ro (C.-I.B.); 2 County Clinical Emergency Hospital of Sibiu, 550245 Sibiu, Romania; raul.stoica10@gmail.com (R.S.); alexandra94.mihai@gmail.com (A.-M.V.M.); 3 Neuroscience Scientific Research Collective, 550082 Sibiu, Romania; 4 “Dr. Gheorghe Preda” Clinical Psychiatry Hospital of Sibiu, 550082 Sibiu, Romania; gandreeamariaps@gmail.com

**Keywords:** anesthesia practices, electroconvulsive therapy, patient safety, patient outcomes

## Abstract

**Background**: Electroconvulsive therapy (ECT) is a well-established treatment for various psychiatric disorders. This retrospective study evaluates anesthesia practices, patient characteristics, and outcomes in ECT over a two-year period at the “Dr. Gheorghe Preda” Clinical Psychiatry Hospital in Sibiu, Romania. **Methods**: From March 2022 to July 2024, the Neuroscience Scientific Research Collective at our institution carried out a retrospective observational study on patients who underwent ECT. The evaluation and treatment protocol involved patients from all over the country. **Results:** The study involved 30 patients aged between 22 and 67 years and a mean age of 39.4 years; among them, 57% were male. The majority of the patients (68%) lived in urban areas, and 80% came from a different county. Schizophrenia was the most prevalent diagnosis (56.6%), followed by depression (40%) and bipolar disorder (3.4%). Common comorbidities included obesity/overweight, high blood pressure, and sinus tachycardia. A total of 330 ECT sessions were conducted, with an average of 11 sessions per patient, and 10 patients underwent multiple treatment courses. The reported adverse events included arterial hypertension, agitation, tachycardia, and shivering. **Conclusions**: This study underlines the safety and effectiveness of ECT when patients are closely monitored. Our results are consistent with the global data, suggesting that ECT is a good treatment option for severe psychiatric conditions with a manageable incidence of adverse events.

## 1. Introduction

Electroconvulsive therapy (ECT) is a psychiatric treatment that dates back to a time when mental illness was not as well understood as currently, and is one of the oldest treatment methods in this field. ECT provided hope to those with severe conditions when options for addressing psychotic disorders were scarce [1,2,3]. Electroconvulsive therapy faced controversy due to historical practices, side effects, media, ethical concerns, variability in practice, and misuse, but its efficacy in alleviating severe depression and psychosis could not be ignored [2,3]. Despite initial concerns, ECT is now an accepted treatment option for mental health disorders, significantly because this technique has become safer and has improved over time [2,3]. Advances in anesthesia care, particularly the development of anesthetic agents and monitoring techniques, have reduced the risks and made the procedure safer and more comfortable for patients. Even though it has been effectively used for almost a century, ECT remains a topic of fascination in the current psychiatric research.

The exact mechanism of action of ECT is not fully understood, but it is believed to involve several neurobiological processes [4]. ECT affects neurotransmitter systems by altering the release, reuptake, and receptor sensitivity of key neurotransmitters like serotonin, norepinephrine, and dopamine [1,4,5]. Studies have shown that ECT enhances neurotransmission and modulates receptors in the brain, similar to the effects observed with antidepressant medications, contributing to improvements in mood, motivation, and cognitive function [1,5,6,7]. In addition to changes in neurotransmitters, ECT promotes neuroplasticity by increasing neurotrophic factors, such as brain-derived neurotrophic factor (BDNF), which are important for synaptic plasticity and neuronal health [1,8]. ECT also enhances inhibitory neurotransmission and has anticonvulsant and anxiolytic effects [1,5,7,9]. Furthermore, it affects the structural and functional connectivity within the brain [5]. Neuroimaging studies have shown changes in the integrity of white matter and functional connectivity in the brain regions involved in mood regulation, suggesting that these connectivity changes may play a role in ECT’s therapeutic effects [1,5,10]. ECT also normalizes the activity of the hypothalamic–pituitary–adrenal (HPA) axis often seen in depression, reducing cortisol levels and potentially alleviating the negative effects of stress hormones on neuroplasticity [1,5,9,11]. Finally, ECT alters cerebral blood flow and regional metabolism [1,5]. It decreases blood flow and glucose metabolism in some cortical regions while increasing circulation in the limbic structures involved in emotional regulation [1,5,12]. This multifaceted action on neurotransmitter systems, neuroplasticity, brain connectivity, and endocrine regulation collectively contributes to the therapeutic efficacy of ECT in treating severe psychiatric disorders. These changes help to restore the balance of neural circuits and improve mental health in patients undergoing ECT.

Electroconvulsive therapy as a nonpharmacologic treatment method has been extensively researched and shown to be a highly effective intervention, particularly in the treatment of depression [13]. A 3-year analysis of life-threatening events in over 3000 electroconvulsive therapy treatment sessions in Germany concluded that ECT is a safe and effective treatment when administered in a controlled and supervised medical setting [14]. Furthermore, ECT has also demonstrated efficacy in addressing conditions such as schizophrenia and other related indications, including catatonia, mania, and treatment-resistant obsessive–compulsive disorder, as well as in certain cases of Parkinson’s disease, among others [13,15,16,17,18,19,20]. ECT is recommended for severe depression that does not respond to antidepressants, and it is a safe and effective treatment option for those experiencing treatment-resistant depression with significant impairment in daily activities [20]. A suitable indication for electroconvulsive therapy is in patients who have shown a positive clinical response to ECT in the past [21]. Nonetheless, ECT quickly alleviates suicidal thoughts with complete resolution observed in a significant percentage of patients (8% of patients after one week, 61% of patients after two weeks, and 81% of patients with the completion of ECT) [15].

Thanks to advancements in technology and modern anesthesia techniques, electroconvulsive therapy is now safer due to administering the treatment within a standard protocol and a controlled medical environment. When administering anesthesia during ECT sessions, the goal is to ensure the patient’s hemodynamic stability, induce amnesia, and facilitate muscle relaxation for effective treatment [22,23,24]. When providing anesthesia, it is important to adhere to the standard guidelines outlined by the American Society of Anesthesiologists (ASA), which regulates the administration of anesthesia, guidelines that also can be applied to ECT treatment [23,25,26,27]. To achieve this, essential monitoring equipment such as a stethoscope, blood pressure monitor, electrocardiography (EKG) monitor, pulse oximeter, suction device, and oxygen delivery system should be easily accessible [23,25,26,27]. Anesthetic induction medications, and ventilatory and resuscitation equipment should also be readily available [23,25,26,27]. To provide even better patient care, supplementary equipment like a nasal cannula or face mask for oxygen administration, bag valve mask, nerve stimulator for assessing neuromuscular blockade, electromyograph (EMG), electroencephalography (EEG) leads, and different-sized blood pressure cuffs should be included as well [23,25,26,27]. Achieving optimal practice in this area necessitates adopting personalized dosing strategies, a comprehensive understanding of patient characteristics, and a multidisciplinary approach [22,23,28].

Administering anesthesia for ECT involves using the lowest effective dose of an induction agent to induce amnesia and minimize drug-induced seizure inhibition [22]. Additionally, muscle relaxants are used liberally to prevent injuries, especially in elderly women with severe osteoporosis [22,29,30]. However, a common challenge in ECT is that the muscle relaxant may last longer than the hypnotic agent, potentially causing paralysis while the patient is awake [22,29,30]. To prevent this, it is recommended to use post-seizure amnestic agents. During the administration of anesthesia for electroconvulsive therapy, anesthesiologists have to maintain vigilance as several potential adverse effects may occur [22,29,30,31]. Cardiovascular complications, such as hypertension, tachycardia, and arrhythmias, can manifest during and after the procedure, particularly for patients with pre-existing cardiac conditions [22,29,32]. Respiratory concerns, including apnea and hypoxemia, require close airway and ventilation management monitoring [22,29]. Common neurological effects, notably confusion, and memory disturbances warrant continuous monitoring [22,29,32]. Furthermore, musculoskeletal considerations encompass the potential for succinylcholine-induced muscle pain, as well as rare occurrences of fractures or dislocations [22,29]. Additionally, metabolic disturbances, mainly electrolyte imbalances caused by psychiatric medication, demand close attention, and the possibility of allergic reactions to anesthetic agents should always be taken into account [22,29]. Comprehensive pre-procedural evaluations and diligent procedural management play critical roles in mitigating these risks and assuring patient safety throughout the course of ECT procedures.

This two-year retrospective study aims to evaluate the anesthesia practices, patient characteristics, and outcomes associated with ECT at our institution. By analyzing the dataset and presenting our protocol for the patients proposed for ECT, we sought to identify patterns that can contribute to the existing knowledge on ECT, provide insights into optimizing anesthesia practices, highlight the importance of personalized treatment approaches, and strengthen the concept of safe ECT.

## 2. Materials and Methods

Over the past two years, we undertook a comprehensive retrospective observational study examining the outcomes of patients who underwent electroconvulsive therapy at our institution. The team at the Neuroscience Scientific Research Collective of the “Dr. Gheorghe Preda” Clinical Psychiatry Hospital in Sibiu, Romania, formulated a protocol for the treatment of patients undergoing ECT. In March 2022, the Electroconvulsive Therapy department started to treat patients from all across the country.

Inclusion criteria: The evaluation committee consists of two senior psychiatrists with experience and training in ECT. They evaluate the case file, considering the anesthetist’s medical opinion and advice. The patient must have a recommendation from the attending psychiatrist, including the case presentation and treatment history, to justify the concept of therapeutic resistance to be eligible for ECT. The pre-anesthetic evaluation is usually performed in our institution after the patient’s admission. It includes a cardiologic consult, neurology consult, and laboratory parameters (blood count, renal and hepatic assessment, ionogram, and coagulation panel). If the patient has an implant (pacemaker, cochlear implant, orthopedic implant, etc.), we require a recommendation from the manufacturer that the implant is ECT-safe. After the initial pre-anesthetic evaluation and assessment, based on clinical judgment, further investigations may be recommended (echocardiography, imagistic investigations, and additional blood work). Informed consent is acquired in compliance with the legal and ethical considerations for individuals diagnosed with mental illnesses, in strict adherence to the regulations of our jurisdiction. Psychological assessments are carried out before, during, and after the treatment course.

Exclusion criteria: Patient’s refusal, patients under 18 years old, signs indicating that the patient is not resistant to the maximal accepted pharmacological treatment, and/or the existence of somatic conditions that pose a high risk to the patient’s life after receiving electroconvulsive therapy (e.g., advanced or end-stage diseases).

The therapy is conducted every other day, totaling up to three times per week, under general anesthesia performed by an anesthesiologist, with muscle relaxation and manual ventilation using an anesthesia machine. Psychiatric medication is withheld 12 h before each procedure. If the patient’s clinical condition requires it, they will receive the smallest possible dose of psychiatric medication to induce an efficient therapeutic seizure. The concurrent medication that the patient takes for chronic disease (other than psychiatric medication) is carefully analyzed. If it does not interact with the ECT and anesthesia, the background medication is usually continued during the therapeutic course. With this approach, the effect of premedication on the induction of convulsive waves is minimal.

From a logistical standpoint, the department consists of three rooms. The first room is the pre-procedural room, where the patient undergoes clinical evaluation and a pre-procedural interview and receives pre-medication before each ECT session. The second room is equipped with the necessary equipment for shock administration, a procedure table; an anesthesia machine; a vital sign monitor; a medication cabinet containing hypnotics, analgesics, muscle relaxants, and resuscitation medications; airway management devices; a secretion aspiration device; a defibrillator; and oxygen delivery. The second room is where the shock administration takes place. The third room is the post-procedural room, where the patient is monitored after the shock administration and after the return of spontaneous breathing. The patients are transferred to the ward approximately 20 min after regaining pre-procedural neurological status and achieving hemodynamic and respiratory stability.

Before each session, a short medical interview is conducted, and intravenous access is established to administer Acetaminophen intravenously for headache prevention. Subsequently, the patient is positioned on the procedure table and monitored for EEG (on ECT machine), ECG (on anesthesia monitor and ECT machine), Spo2 (on anesthesia monitor), motor activity (OMS—optical motion sensor) during the treatment (on ECT machine), and arterial blood pressure (on anesthesia monitor). Following the verification of all equipment and medication, the team comprising an anesthetist, psychiatrist, nurse, and patient provides in unanimity the consent to start the procedure. Simultaneously with preoxygenation via an induction mask, the nurse administers atropine to prevent bradycardia and excessive salivation post-electroconvulsive therapy. During the induction phase, Propofol is administered to achieve the loss of consciousness (the smallest dose possible to avoid awareness but to have a minimum influence on convulsive waves), along with Succinylcholine for muscle relaxation. The patient is hyperventilated (to lower the convulsive threshold) using an induction mask via an anesthesia machine. Following the cessation of fasciculations, the psychiatrist verifies one more time the ECT dose and administers the electrical shock. Once the seizures cease and no further convulsive activity is present on the EEG, the patient is ventilated until spontaneous ventilation resumes. Subsequently, the patient is transferred to the post-intervention area and administered O_2_ via facemask and 20% Mannitol. Complications that occur after the procedure (e.g., tachycardia and hypertension) are treated with specific medications for the adverse event in question (e.g., intravenous metoprolol for tachycardia and intravenous urapidil for hypertension).

Vital signs are continuously monitored, and after the patient regains pre-procedural neurological status and achieves hemodynamic and respiratory stability for 20 min, they are transferred to the ward.

The collected data included the patient’s age, gender, geographical origin, diagnosis, comorbidities, the number of ECT sessions per patient, treatment courses per patient, and adverse events requiring specific treatment after ECT. The data were collected from the patient’s medical records and analyzed manually.

The study was approved by the Institutional Research Ethics Committee of the “Dr. Gheorghe Preda” Clinical Psychiatry Hospital of Sibiu (approval no 11326/01.08.2024) and complied with the Declaration of Helsinki.

## 3. Results

**Figure 1:**
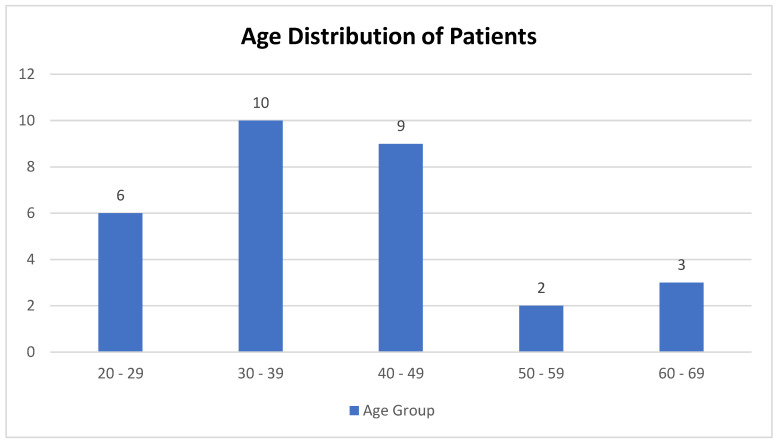
Age distribution of patients.

In this retrospective study, a cohort of 30 patients ranging in age from 22 to 67 years (with a mean age of 39.4 years) was examined. The majority of the patients (n = 10) were within the 30–39 age group, followed by the 40–49 (n = 9) and 20–29 (n = 6) age groups (Figure 1).

**Figure 2:**
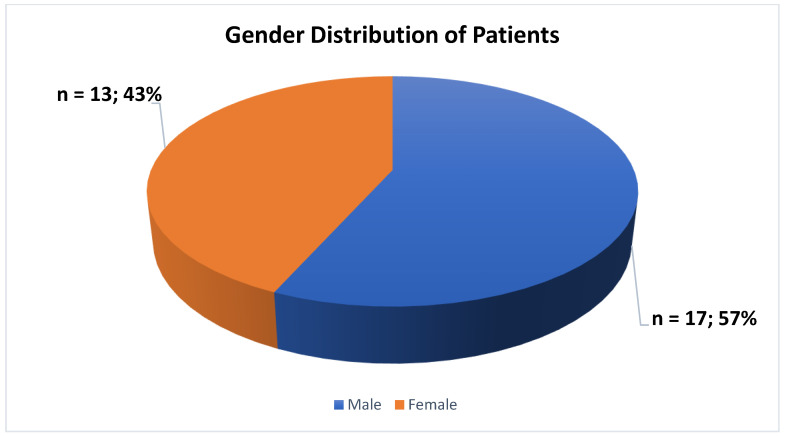
Gender distribution of patients.

The majority of the participants involved in the current investigation were male, constituting 57% (n = 17), while 43% (n = 13) were female (Figure 2).

**Figure 3:**
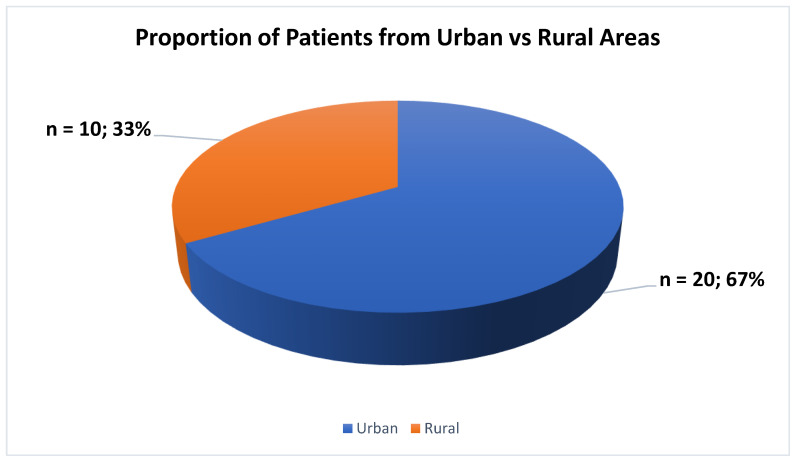
Proportion of patients from urban vs. rural areas.

The majority of the patients, specifically n = 20 (67%), were found to be residing in urban areas, while n = 10 (33%) were living in rural areas (Figure 3).

**Figure 4:**
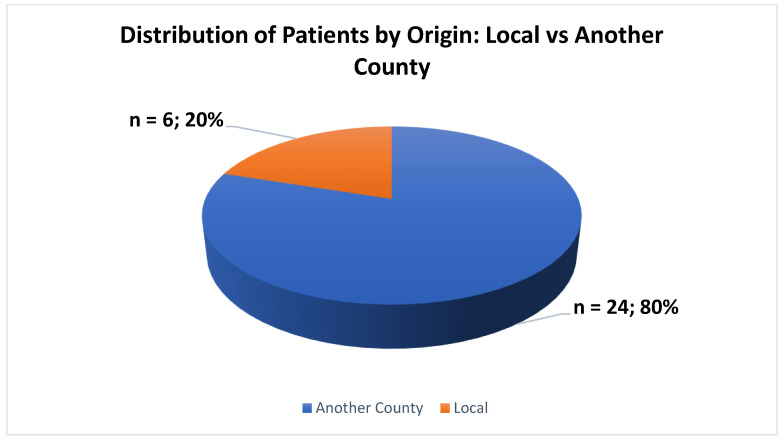
Distribution of patients by origin.

The patient’s demographics revealed that the predominant proportion, comprising n = 24 (80%), originated from a different county, while a smaller cohort, totaling n = 6 (20%), represented individuals who originated from the county in which the hospital was situated (Figure 4).

**Figure 5:**
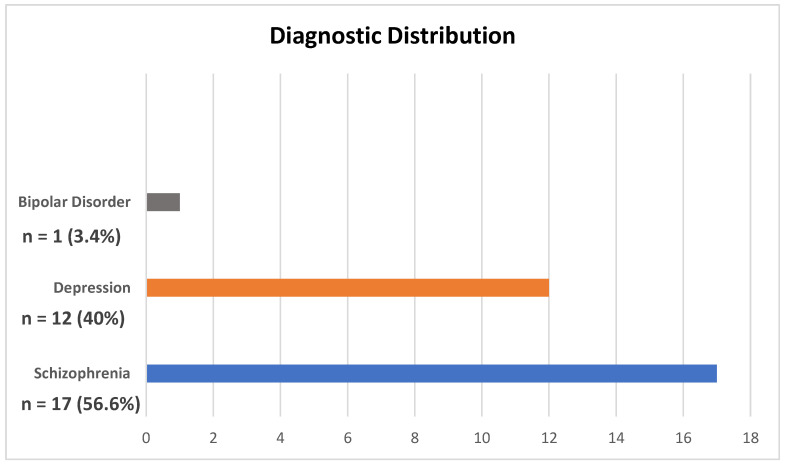
Diagnostic distribution.

The results indicate that a significant proportion of the patient population, specifically n = 17 (56.6%), were diagnosed with schizophrenia. Additionally, n = 12 (40%) of the patients were diagnosed with depression, followed by a smaller proportion of n = 1 (3.4%) diagnosed with bipolar disorder (Figure 5).

**Figure 6:**
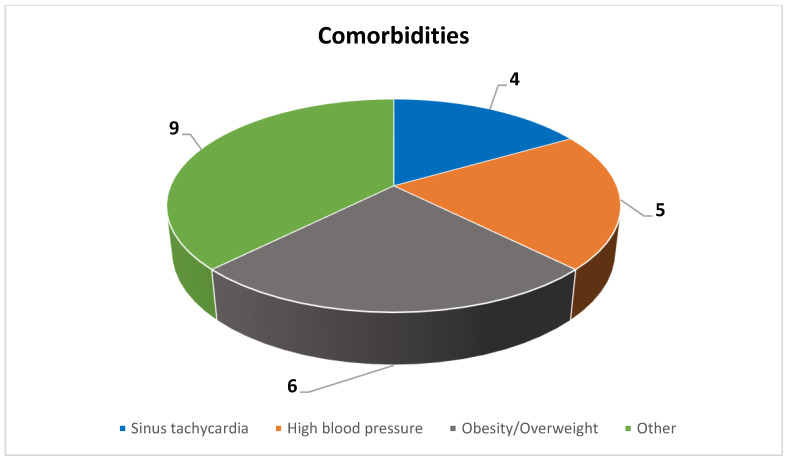
Comorbidities.

The most prevalent comorbidities observed among the study participants included obesity/overweight (n = 6), followed by high blood pressure (n = 5) and sinus tachycardia (n = 4). Additionally, other comorbidities identified in the study cohort comprised hypothyroidism (n = 1), mild intellectual disability (n = 1), a history of substance abuse (n = 1), a history of head injury (n = 1), a history of abdominal surgery (n = 1), glaucoma (n = 1), post-COVID-19 syndrome (n = 1), cortical atrophy (n = 1), and stenosis of the carotid artery (n = 1) (Figure 6).

**Figure 7:**
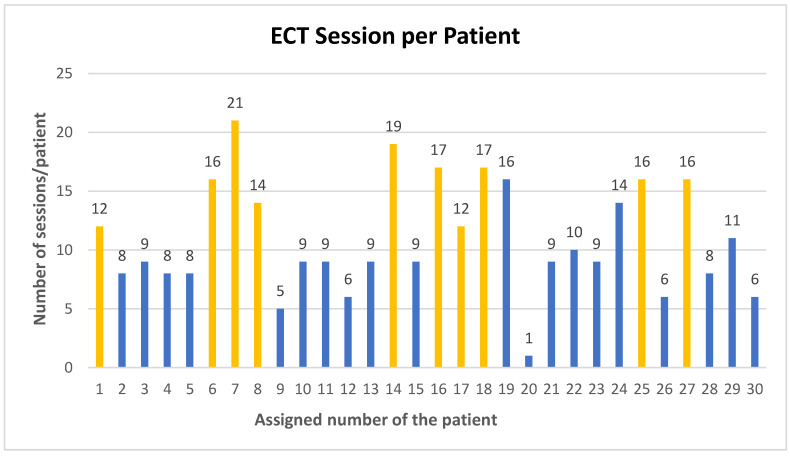
ECT session per patient (the yellow columns represent the patients who had more than one treatment course, and the blue columns represent the patients who had one treatment course).

The analysis revealed a total of three hundred and thirty ECT sessions conducted in our institution (from March 2022 to July 2024). Ten patients were readmitted for maintenance electroconvulsive therapy and underwent more than one treatment course, as denoted by the yellow columns, while twenty patients received a single treatment course, represented by the blue columns. Further analysis revealed that, on average, each patient underwent eleven ECT sessions, with the highest number of sessions recorded being twenty-one sessions per patient and the lowest, one session per patient (Figure 7).

**Figure 8:**
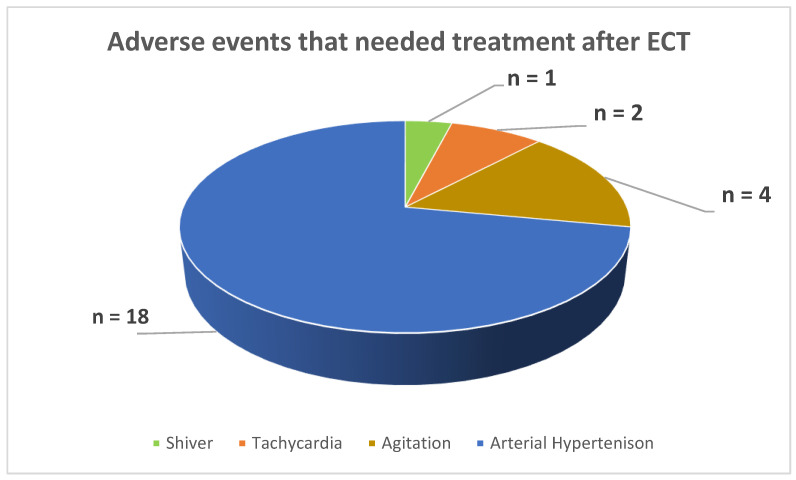
Adverse events that needed treatment after ECT.

From the 330 ECT sessions, the adverse events at the end of the procedure that needed pharmacological treatment were as follows: an incidence of n = 14 (4.24% of the total sessions) of arterial hypertension, n = 4 (1.21% of the total sessions) of agitation, n = 2 (0.66% of the total sessions) of tachycardia, and n = 1 (0.30% of the total sessions) experiencing shivering (Figure 8).

## 4. Discussion

Our findings indicate that the distribution of the patients undergoing electroconvulsive therapy at our institution was in the 30–39 (n = 10) age group, followed by 40–49 (n = 9) with an average of 39.4 years. This observation aligns with the existing literature, where the average age of younger patients is reported to be 46.2 years [33,34,35]. The significant presence of younger patients may indicate a trend toward earlier intervention in psychiatric illnesses, emphasizing the importance of understanding demographic nuances in the ECT patient population.

In our investigation, a significant number of the participants were male, making up 57% (n = 17), while 43% (n = 13) were female. The gender distribution in the existing literature is heterogeneous. In some studies, the female proportion is greater, while in others, the male proportion is higher. It is worth noting, however, that the difference between the number of male and female patients treated with ECT reported in the literature is small. Our results indicate a slight male predominance. Plausible explanations for this trend can be the type of diagnosis (more schizophrenia cases vs. fewer depression cases), disease severity, comorbidities, societal attitudes toward mental health and treatment, and local referral practices. While disparities in the use of electroconvulsive therapy based on gender exist worldwide, it is important to conduct additional research to fully comprehend the underlying factors contributing to these differences [33,34,35,36].

Our data reveal that a significant proportion of the patient cohort, specifically n = 21 (68%), were found to be residing in urban areas, while n = 10 (32%) were identified as living in rural areas. The observed distribution aligns with the prior literature findings that have highlighted the same predominance of urban residency among ECT patients. The urban–rural divergence in ECT can be attributed to the differential access to mental health facilities, greater availability, and greater public medical education, awareness, and acceptance within urban populations regarding specialized psychiatric treatment, including ECT utilization rates. The patients in rural areas face many challenges, such as long travel distances to healthcare facilities, limited transportation options, a lack of mental health services, financial barriers, difficulties with patient follow-up, and increased stigma associated with mental health and ECT in rural communities. These challenges can make it harder for the patients to seek treatment. To address these issues, it is important to improve rural healthcare infrastructure, expand telemedicine and telepsychiatry services, and encourage mental health professionals to work in rural areas [34,37,38,39].

The patient demographic data showed that 14 out of 20 patients (70%) were from a different county, while the remaining 6 patients (30%) were from the same county as the hospital. This indicates that our hospital is a regional center for specialized treatments such as ECT. Our well-established patient care pathways, good collaboration between medical centers, and referral networks attract patients from other parts of the country. These findings highlight significant gaps in the availability and accessibility of ECT in neighboring counties, indicating a need for a broader distribution of specialized mental health services.

The results from our analysis indicate that a significant proportion of the patient population, comprising a total of n = 17 (56.6%), were diagnosed with schizophrenia. Furthermore, n = 12 (40%) of the patients were diagnosed with depression, followed by a smaller proportion of n = 1 (3.4%) diagnosed with bipolar disorder. Our study observed that the diagnostic indications for ECT align with the existing literature, which consistently reports a high prevalence of depression as an indication for ECT. Understanding the distribution of diagnoses can aid in resource planning, ensuring that sufficient support and specialized care are available for patients with schizophrenia, depression, and bipolar disorder [33,34,35,38,40,41].

The participants from our study had various comorbidities, with the most common being obesity/overweight (n = 6), followed by high blood pressure (n = 5) and sinus tachycardia (n = 4). The other comorbidities observed in the study cohort included hypothyroidism, mild intellectual disability, a history of substance abuse, a history of head injury, a history of abdominal surgery, glaucoma, post-COVID-19 syndrome, cortical atrophy, and stenosis of the carotid artery. There is a well-established connection between having a psychiatric diagnosis and being overweight, and the level of serum lipid can affect the effectiveness of ECT. These findings highlight the need for an interdisciplinary and personalized approach to managing patients undergoing ECT, especially considering the prevalence of comorbid conditions such as obesity, high blood pressure, and sinus tachycardia [42].

From March 2022 to July 2024, our institution conducted 330 ECT sessions. Ten patients were readmitted for maintenance electroconvulsive therapy and underwent more than one treatment course, while twenty received a single treatment course. The fact that ten patients were readmitted for maintenance ECT and underwent more than one treatment course suggests that there is a subgroup of patients who may need ongoing treatment to maintain the therapeutic benefits. This highlights the importance of personalized and continuous care plans for certain individuals. Upon further analysis, it was found that on average, each patient underwent eleven ECT sessions. The highest number of sessions recorded was twenty-one per patient, and the lowest was one session per patient due to the patient’s refusal to proceed with the treatment. The number of electroconvulsive therapy (ECT) sessions given to patients worldwide varies significantly, ranging from 1 to 22 sessions. The existing research indicates that the average number of sessions typically falls between 5 and 11, which is consistent with our observations. The range of 1 to 21 sessions at our institution is comparable to the global range of 1 to 22 sessions. This suggests that our institution’s practices are within the typical ECT applications worldwide [34,40,43,44].

During the 330 ECT sessions conducted at our institution, the following adverse events occurred at the end of the procedure, requiring pharmacological treatment in the procedure room: 14 cases (4.24% of the total sessions) of arterial hypertension, 4 cases (1.21% of the total sessions) of agitation, 2 cases (0.66% of the total sessions) of tachycardia, and 1 case (0.30% of the total sessions) of experiencing shivering. The low occurrence of adverse events aligns with the reports from the literature and is interpreted as manageable issues when patients are carefully monitored, and appropriate pharmacological treatments are available. Based on the majority of studies, electroconvulsive therapy (ECT) is generally regarded as safe when patients are carefully monitored. The most commonly reported adverse events are temporary high blood pressure, arrhythmias, and cognitive impairment [45,46,47,48,49,50,51].

## 5. Conclusions

ECT remains an important therapeutic option for patients with severe psychiatric conditions when other treatment approaches have been ineffective. This two-year retrospective study offers insights into the anesthesia practices, patient characteristics, and outcomes associated with electroconvulsive therapy at our institution. The results of our analysis confirm the safety and effectiveness of ECT as a treatment for severe psychiatric disorders when patients are carefully monitored and prepared for the procedure. The protocols and practices developed and implemented at our institution have shown a high level of safety and effectiveness and can be used in similar medical settings. The continuous research and refinement of ECT protocols are essential to further improve patient outcomes and minimize adverse events.

## Data Availability

Data are contained within this article.
